# Large-scale Discovery of Substrates of the Human Kinome

**DOI:** 10.1038/s41598-019-46385-4

**Published:** 2019-07-19

**Authors:** Naoyuki Sugiyama, Haruna Imamura, Yasushi Ishihama

**Affiliations:** Graduate School of Pharmaceutical Sciences, Kyoto University, Sakyo-ku, Kyoto 606-8501 Japan

**Keywords:** Proteomic analysis, Proteomics

## Abstract

Kinase networks are important for cellular signal transduction. Despite tremendous efforts to uncover these signaling pathways, huge numbers of uncharacterized phosphosites still remain in the human proteome. Because of the transient nature of kinase-substrate interactions *in vivo*, it is almost impossible to identify direct substrates. Here, we present a strategy for the rapid, accurate and high-throughput discovery of *in vitro* kinase substrates using quantitative proteomics. Using 385 purified kinases (354 wild-type protein kinases, 21 mutants and 10 lipid kinases), we identified a total of 175,574 potential direct kinase substrates. In addition, we identified novel kinase groups, such as one group containing 30 threonine-directed kinases and another containing 15 serine/threonine/tyrosine kinases. Surprisingly, we observed that the diversity of substrates for tyrosine kinases was much higher than that for serine-threonine kinases.

## Introduction

Reversible protein phosphorylation catalyzed by both protein kinases and phosphatases plays essential roles in cellular signal transduction that controls a variety of cellular functions^1^. Based on the results from human genome project, at least 518 protein kinases are considered to be encoded in human genome^2^, and at least 70% of all human proteins are phosphorylated by these kinases^3,4^. This indicates that each protein kinase involves roughly hundreds of phosphorylation events, and the phosphorylation network formed by them must be extremely complicated.

It is well known that kinase-mediated phosphorylation signals are involved in various diseases such as cancer, and often cause the disease itself or drive its progression. Large-scale cancer genome sequencing projects have been conducted to detect mutationally activated proteins, including kinases, in cancer signaling pathways^5^, and inhibitors of oncogenic kinases have been extensively developed for cancer therapy since the success of imatinib for Bcr-Abl-positive chronic myeloid leukemia (CML) patients. However, although genomics- and proteomics-based approaches have been applied to characterize kinases^6–8^, we still lack basic information on many kinases, such as their substrates, phosphorylation motifs and molecular functions, and more information is required to unveil the entire network of protein phosphorylation.

In recent years, mass spectrometry (MS)-based phosphoproteomics have been improved, especially phosphopeptide enrichment approaches, such as immunoprecipitation with anti-phosphotyrosine antibodies^9^, immobilized metal ion affinity chromatography (IMAC)^10,11^ and metal oxide chromatography with enhancers^12–15^. Many biological applications of these technologies have been reported, e.g., to study the signal transduction of epidermal growth factor^16^, cell cycle regulation^3,17^, embryonic stem cell differentiation^18^ and tissue-specific phosphorylation events^19^, resulting in the identification/quantification of tens of thousands of phosphosites. Thus far, over 100,000 phosphosites in the human proteome have been registered in public databases such as UniProtKB^20^, PhosphoELM^21^, PhosphoSitePlus^22^, PHOSIDA^23^ and HPRD^24^. Although recent advances in MS-based phosphoproteomics have enabled identification of more than 10,000 sites by a single LC/MS/MS measurement^25^, the nature of the exact kinases responsible for these sites largely remains to be elucidated. Moreover, even at the protein level, less than 40% of phosphoproteins can be mapped to the KEGG pathway database^26^, and the functional roles of the phosphorylated sites are still unclear in many cases^27^.

It is considered that in general each kinase prefers to phosphorylate the substrates with particular sequence, namely, a phosphorylation motif, which often has been utilized for *in silico* prediction of kinase-substrate relationships. On the basis of limited dataset of *in vitro* kinase assay, various computational tools for prediction of responsible kinases or potential substrates, such as Scansite^28^ and NetPhorest^29^, have been developed to date. In some cases, public datasets on protein-protein interaction have been integrated in some computational tools for kinase prediction from queried phosphosites^30–32^. Many *in silico* prediction tools for kinase-substrate relationships have been developed^32,33^, however, a bottleneck in these tools is the absence of a sufficient experimental kinase-substrate dataset to provide phosphorylation motifs of an individual protein kinase.

Recently, peptide arrays using a synthetic peptide library with randomized sequences^34^ or a position scanning peptide library^35^ have been employed to extract phosphorylation motifs rapidly^36–38^. Mok *et al*. have reported large-scale analysis of yeast phosphorylation motifs using peptide library screening and identified motifs for 61 kinases^39^. Newman *et al*. have reported 300 phosphorylation motifs for 289 kinases by *in vitro* kinase profiling using a protein array, although phosphosites were not experimentally identified^40^. These array-based approaches allow a high-throughput profiling of substrate preference for each kinase in a large scale. Regarding MS-based approaches with a biological sample as a substrate source, Huang *et al*. identified PKA and PKG substrates from rat uteri by using an *in vitro* kinase assay followed by a phosphoproteomics approach^41^; 61 and 12 *in vitro* substrates as well as consensus phosphorylation motifs were found for PKA and PKG, respectively, and found that some of the *in vitro* substrates were potential physiological substrates. Zhang *et al*. also reported a solid-phase approach for the identification of *in vitro* CK2 substrates using immobilized proteins from a cell extract as a substrate source^42^, and suggested the method may reduce effects of intrinsic active kinases compared with solution-based approach. Kettenbach *et al*.^43^ and Douglas *et al*.^44^ investigated *in vitro* kinase substrates using digested peptides from a cell lysate as a substrate source and identified hundreds to thousands of *in vitro* substrates of each kinase. Furthermore combination use of *in vitro* assay and *in vivo* phosphoproteome analysis was utilized to identify physiologically relevant substrates^45,46^. MS-based *in vitro* kinase assays became a powerful tool, nevertheless the total number of human kinases employed thus far in the individual study is still quite limited (less than 15).

Previously, we developed an LC-MS/MS-based *in vitro* kinase assay to profile three human protein kinases, Erk1, AKT1 and PKA, in which dephosphorylated lysate proteins were used as the substrate source for *in vitro* kinase reaction and the phosphorylated proteins were digested by trypsin, fractionated by ion-exchange StageTip, enriched by titania chromatography and analyzed by LC-MS/MS^47^. Thousands of *in vitro* phosphorylation sites for each kinase were successfully identified and several new phosphorylation motifs together with the known motifs reflecting the specificity of each kinase were found. In this study, we modified the previous approach for kinome-wide applications, in which a stable isotope-based quantitation approach was used through the study to reject the endogenously phosphorylated proteins remaining in the substrate source even after phosphatase-treatment. By using this approach, 385 human recombinant kinases consisting of 354 wild-type protein kinases, 21 mutants and 10 lipid kinases were analyzed to acquire an *in vitro* substrate library as well as phosphorylation motifs. Substrate preference of each kinase was more accurately obtained by using quantitative phosphoproteomic approach with an isotopical labeling to reject the contaminant phosphoproteins constantly through this study. Using these results, a novel ‘kinome tree’ based on kinase-substrate relationships was proposed.

## Results

### Large-scale identification of *in vitro* kinase substrates

**Figure 1:**
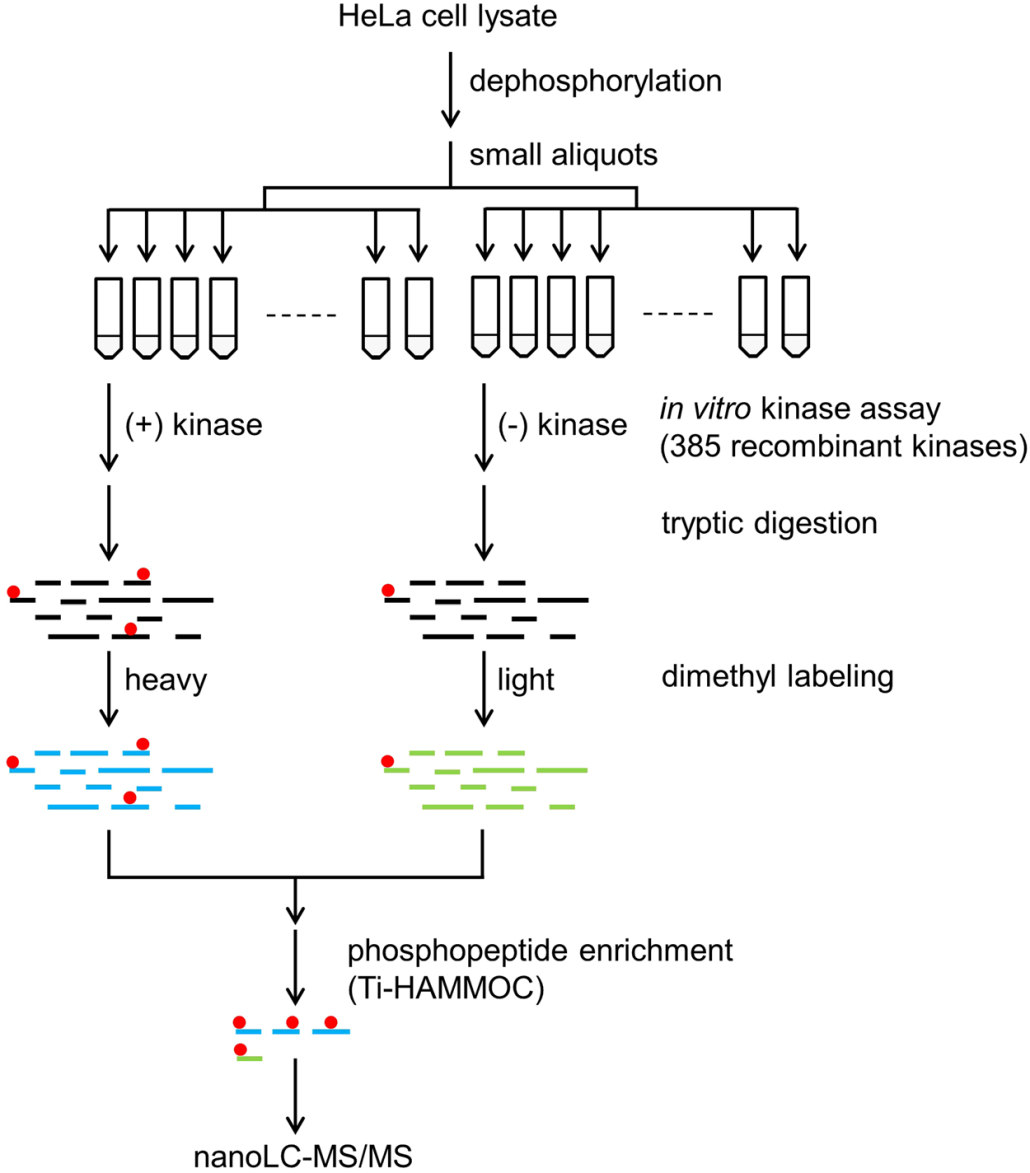
Overview of *in vitro* kinase profiling. HeLa cell lysate was dephosphorylated with alkaline phosphatase, and spiked phosphatase was inactivated with heat. Small aliquots of the dephosphorylated lysate were reacted with recombinant protein kinase. The mock-reacted sample was used as a control. After tryptic digestion, both samples were labeled with heavy or light formaldehyde. Phosphopeptides enriched with titania-HAMMOC^14^ were analyzed with nanoLC-MS.

Using dephosphorylated HeLa cell lysate as the substrate source, 385 kinds of recombinant human protein kinases were individually employed for an *in vitro* kinase assay (Fig. 1). Prior to the kinase assay, we examined the dephosphorylation efficiency of thermo-sensitive alkaline phosphatase (TSAP). As shown in Supplementary Fig. S1, most of the phosphosites were dephosphorylated at an efficiency of over 95%. However, a small population of phosphosites was not effectively dephosphorylated by TSAP, indicating that it is necessary to quantify the residual phosphopeptides to identify kinase substrates accurately. In this study, chemical dimethyl labeling of peptide amino groups^48^ was carried out with exactly the same materials used for kinase-treated and control samples after cell lysis and TSAP dephosphorylation (Supplementary Fig. S1). We used a peptide peak area ratio of kinase-treated to control as an acceptance criterion for determining kinase substrate, where the ratio should be more than 2. False-positive substrates derived from inherent phosphopeptides which were not able to be dephosphorylated by TSAP were successfully rejected by using the quantitative analysis with the isotopic labeling.

A reproducibility of *in vitro* kinase assay was evaluated by comparison between biological and technical triplicates (Supplementary Fig. S2). Distribution of overlaps of identified phosphopeptides between each pair of biological replicates was similar to that observed in technical replicates. Note that a reproducibility of quantification with dimethyl labeling was also confirmed by RSD of the peak area ratio of kinase-treated to control (9.5 ± 15.8% in biological triplicates).Activities of endogenous protein kinases in the cell lysate were estimated by selected reaction monitoring (SRM) of kinase-activity targeted phosphopeptides^49^. Phosphorylation levels of activity-regulatory sites of the kinases belong to EGFR-MAPK pathway were not significantly changed by spiking recombinant EGFR (Supplementary Fig. S3). This result suggested that endogenous kinases were not activated by a spiked recombinant kinase during the *in vitro* assay.

**Figure 2:**
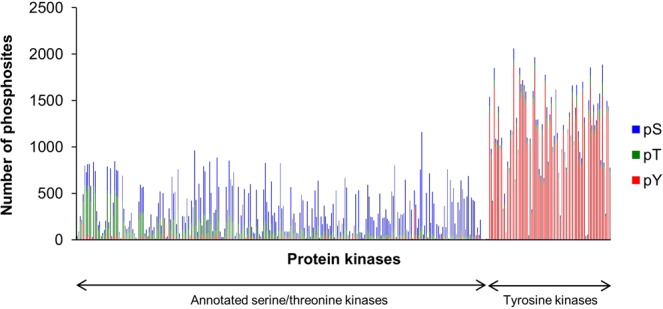
pS/T/Y distribution of *in vitro* substrates for STK and TK. Phosphorylated STY ratios are indicated for 354 wild-type human protein kinases, including 81 TKs and 273 STKs. The blue, green and yellow bars represent pS, pT and pY, respectively. Phosphosites were confirmed by (**a**) the presence of site-determining ions or (**b**) localization probability P > 0.75 based on PTM score.

An *in vitro* kinase assay was performed for 354 wild-type recombinant kinases. In total, 29,837 non-redundant phosphosites were identified. The use of the quantitative criteria mentioned above left 20,669 reliable phosphosites, based on 175,574 kinase-substrate pairs (Table 1, Supplementary Tables S1 and S2). There were 9,998 phosphorylated serine (pS), 4,698 phosphorylated threonine (pT) and 5,973 phosphorylated tyrosine (pY) residues in total. The average number of substrates per kinase was 201 serines (66%), 85 threonines (28%) and 18 tyrosines (6%) for serine/threonine protein kinases (STKs) and 64 serines (6%), 44 threonines (4%) and 1,036 tyrosines (91%) for tyrosine protein kinases (TKs) (Fig. 2). The high selectivity in amino acid recognition suggested that most of the identified substrates were directly phosphorylated with a spiked kinase rather than an endogenous kinase activated by the spiked one. In this study, most of the endogenous kinases should have been inactivated by TSAP dephosphorylation and/or heat denaturation before the kinase reaction. In addition, subcellular localization and protein complexes were corrupted during cell lysis and denaturation. Further, the concentrations of the kinases and substrates were highly diluted relative to the concentrations under physiological conditions. Therefore, it is unlikely that secondary reactions with endogenous kinase(s) activated by the spiked recombinant kinase occurred under our conditions as evaluated by monitoring activities of endogenous kinases (Supplementary Fig. S3). The possibility of autophosphorylation or phosphorylation by constitutively active kinases was ruled out by applying the criteria shown in Supplementary Fig. S1. A small percentage of pY was identified in the assay for STKs, and pS and pT were identified for TKs, even after more stringent filtering based on PTM scores^16^ (See Supplementary Table S1). This is presumably because the amino acid preferences of STKs and TKs are not highly exclusive under *in vitro* conditions. Kettenbach *et al*. recently reported that Haspin showed slight tyrosine-phosphorylation activity in an *in vitro* assay using motif-containing synthetic peptides, suggesting that slightly phosphorylated tyrosines of abundant proteins might be detectable as STK substrates. Another reason is that a portion of the phosphorylated tyrosines were derived from spiked STKs themselves, and these had been prepared as commercial products to maximize their kinase activity. The phosphorylation statuses of the tested kinases were investigated individually by LC-MS analysis of their tryptic digests. We found that 87% of the tested TKs and 23% of the STKs had at least one phosphorylated serine/threonine and tyrosine, respectively. In addition, it is still difficult to correctly determine phosphosite localization from incompletely fragmented MS/MS spectra, even though some probability-based scoring methods for site determination, such as Ascore^50^ and PTM score, have been developed.

**Table 1:** *In vitro* kinase substrates for 354 wild-type human protein kinases.

	Number of phosphosites^a^			
	pS/T/Y	pS	pT	pY
All kinases	175,574	59,945	26,724	88,905
STKs	83,018	54,945	23,160	4,913
TKs	92,556	5,000	3,564	83,992
Unique phosphosites	20,669	9,998	4,698	5,973

^a^The phosphosites were obtained from identified phosphopeptides that satisfied quantitative criteria, *i*.*e*., a peak area ratio of more than 2.

### Identification of phosphorylation motifs

Based on the *in vitro* substrate source consisting of 175,574 substrates with 354 wild-type protein kinases, phosphorylation motifs for individual TKs or STKs were extracted using motif-x^51^. By merging the data for all kinases, we obtained a total of 1,427 phosphorylation motifs targeted by 289 kinases (See Supplementary Table S3). To our knowledge, this is the largest list of motif-kinase relationships reported so far. Most of the extracted phosphorylation motifs were not structurally rigid. The maximum number of fixed amino acids, including phosphorylated S/T/Y, in the extracted phosphorylation motifs was 4, and the numbers of phosphorylation motifs consisting of 2, 3 and 4 fixed amino acids were 1,219 (85.4%), 204 (14.3%) and 4 (0.3%), respectively. On average, 4.12 and 7.28 phosphorylation motifs were extracted per STK and TK, respectively. The STK motifs could be categorized into four groups, *i*.*e*., hydrophobic, basic, acidic and proline-rich motifs. Among 881 phosphorylation motifs of STKs, 227 motifs contained at least one acidic amino acid, 349 motifs contained a basic amino acid, and 110 motifs contained proline. The average fold-increase value, calculated from foreground and background hits by motif-x as a measure of the significance of the extracted motif^51^, was 11.07 for STK motifs. Meanwhile, the phosphorylation motifs of most TKs were acidic amino acid-rich. Among 546 phosphorylation motifs of TKs, 459 motifs contained acidic amino acids, and there was no proline-containing motif. Most tyrosine kinases showed low specificity, as indicated by the low averaged fold-increase for TK motifs (2.46).

**Figure 3:**
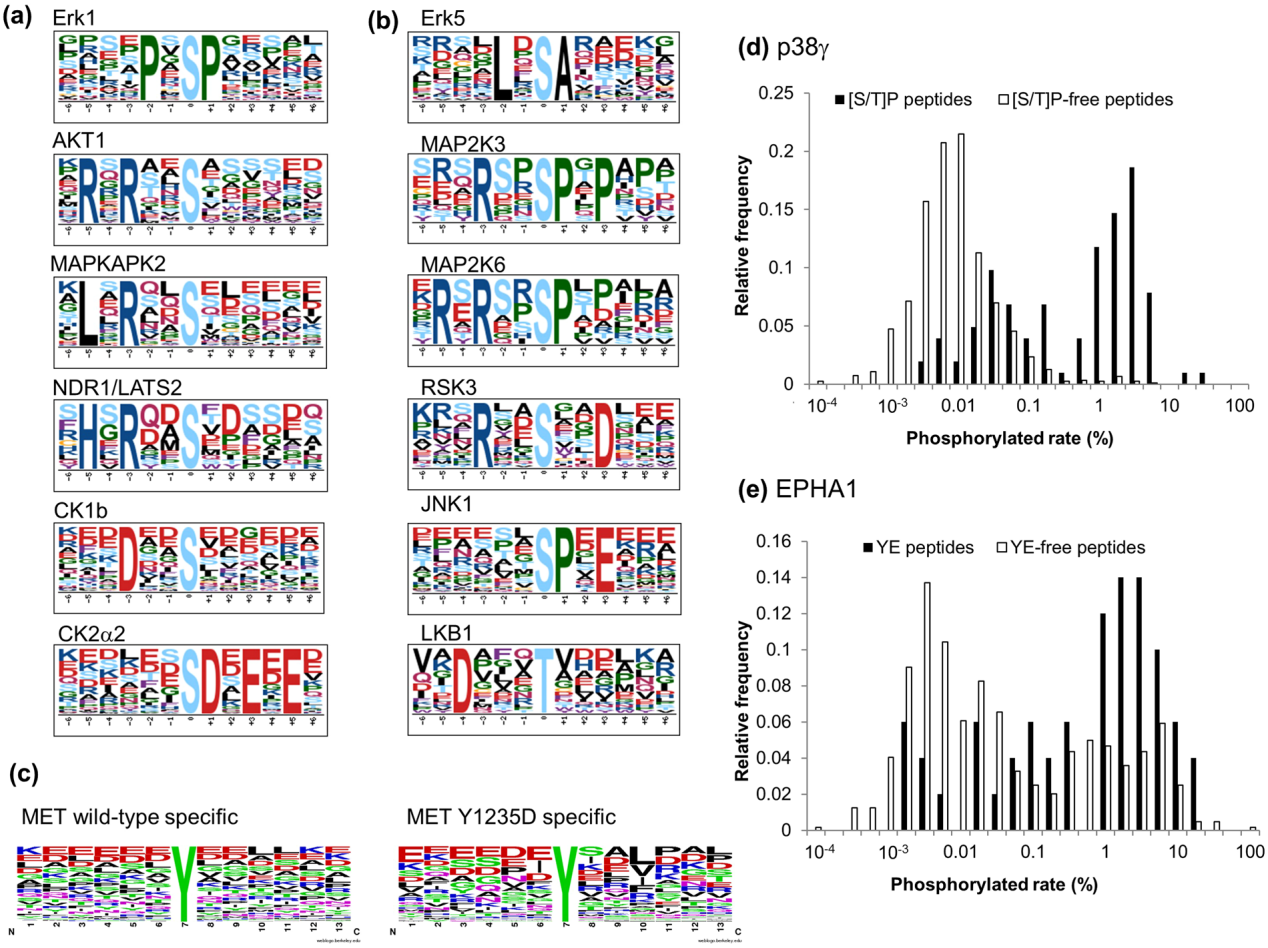
Selected phosphorylation motifs found in this study. (**a**) Known and (**b**) unreported phosphorylation motifs extracted by motif-x with a significant score from the *in vitro* kinase substrates (see Supplementary Table S3 for all motifs covering 306 kinases, 12 families and 5 clusters). (**c**) Amino acid frequency in specific substrates for wild-type or Y1235D mutant of MET. These logos were generated by using Weblogo^74^. (**d**,**e**) Correlation between phosphorylation motif and phosphorylation stoichiometry. Phosphorylation stoichiometry was obtained from the peak area ratio of the corresponding dephosphorylated sample after *in vitro* kinase reaction with (**d**) p38γ and (**e**) EPHA1 (as described in Supplementary Information).

Typical examples of extracted phosphorylation motifs are shown in Fig. 3. The kinases in the CMGC group, which mainly included cyclin-dependent kinases (CDK) and mitogen-activated protein kinases (MAPK), had pS/T-P-containing motifs, like Erk1. From the CAMK group (calmodulin–regulated kinases) and AGC group (consisting of cyclic-nucleotide and calcium-phospholipid-dependent kinases, ribosomal S6 kinases and G-protein-coupled kinases), basic amino acid-rich motifs were obtained, as seen in the Akt1, MAPKAPK2 and NDR1 motifs. Usually, arginine and lysine appeared in the motif sequences for CAMK and AGC, although the motifs of the NDR and LATS families contained a histidine residue at the −5 position, as previously reported^52^. The casein kinase 1 (CK1) and 2 (CK2) families and some other kinases showed phosphorylation of peptides containing D/E-rich motifs.

Some protein kinases showed ‘unreported’ phosphorylation motifs that are different from already-known motifs (Fig. 3(b)), for example, Erk1, 2 and 5 phosphorylated sites containing alanine at the +1 position as well as the known motifs P-X-p(S/T)-P and p(S/T)-P. In the case of dual-specificity MAP2Ks, the known phosphorylation motif pT-X-pY was not extracted, although single phosphorylation of pT-X-Y-containing peptides was observed. The most common sequence observed in phosphorylation motifs of the MAP2K series was R-X-X-pS-P, which was also found to be a major phosphorylation motif of the dual-specificity kinases of the DYRK family.

### Profiling of mutant protein kinases and lipid kinases

The *in vitro* substrates of 21 tumor-associated mutant protein kinases and 10 lipid kinases (LKs) were also investigated in the same manner as described for the wild-type kinases. Tested LKs phosphorylated few proteinous substrates, and no significant phosphorylation motifs could be extracted (See Supplementary Tables S1 and S2).

In general, extracted phosphorylation motifs of mutant kinases were similar to those of wild-type kinases, though the MET mutant Y1235D had a different phosphorylation motif, pY-X-X-R (Fig. 3(c)). Considering that the number of substrates phosphorylated by wild-type MET was approximately 3 times higher than that of the Y1235D mutant, the Y1235D mutant showed weaker kinase activity than wild-type MET, as reported^53,54^. Although 74% of the substrates targeted by the mutant were also phosphorylated by wild-type MET, more than half of the mutant-specific substrates had leucine, isoleucine, valine or phenylalanine at the +3 position. The Y1235D mutant showed a significantly different specificity, given that only approximately 30% of substrates phosphorylated by wild-type MET had such a hydrophobic amino acid at the +3 position.

### Determination of phosphorylation stoichiometry

Phosphorylation stoichiometry or occupancy of the *in vitro* kinase reaction was determined by measuring phosphopeptides and the corresponding non-phosphopeptides. As a test sample, we investigated the phosphorylation stoichiometry of each substrate peptide for the serine/threonine protein kinase p38γ and tyrosine kinase EPHA1 to confirm the significance of the phosphorylation motifs (Fig. 3(d)). There were two populations in the distribution of phosphorylation stoichiometry for both kinases. The peptides that were phosphorylated at a high rate by p38γ almost exclusively contained pS-P or pT-P, which were identified as phosphorylation motifs by the *in vitro* assay and are known consensus motifs of MAP kinases, although not all pS/T-P-containing peptides were phosphorylated with high stoichiometry. In the case of EPHA1, phosphopeptides containing the motif pY-E tended to have higher phosphorylation relative to peptides lacking pY-E peptides, although the distribution was not exclusive (Fig. 3(e)). These results indicated that the phosphorylation stoichiometry somehow reflected the specificity of kinases and their phosphorylation motifs, but it is difficult to use the significance or fold-change to estimate the phosphorylation stoichiometry.

### Classification of kinases based on substrate peptides

**Figure 4:**
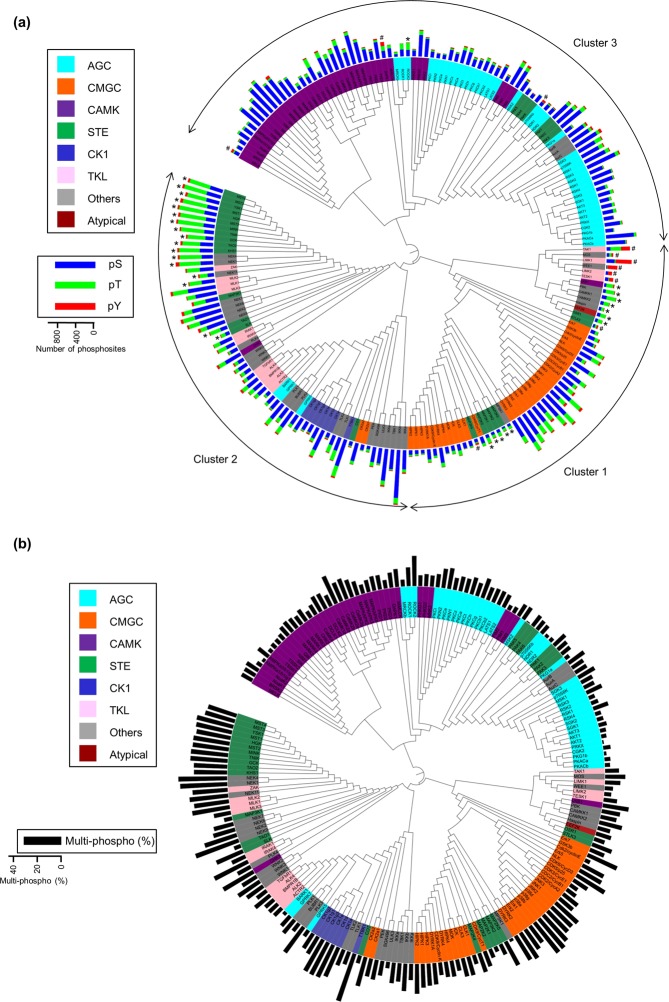
Classification of serine/threonine kinases based on *in vitr*o substrates. A phylogenic kinome tree based on the cluster analysis. The background color of the kinase name indicates the reported kinase group^2^ (see Supplementary Fig. S4 for TKs data). The threonine-specific kinases, *i*.*e*., those for which more than 50% of the substrate sites contain threonine, are indicated by asterisks (*). The kinases indicated by sharps (#) also phosphorylated tyrosine residues at a rate of more than 20% of their total substrates. The bars indicate (**a**) the number of serines/threonines/tyrosines or (**b**) the ratio of multiply phosphorylated peptides identified in each *in vitro* kinase profiling. The maximum ratio of multiply phosphorylated peptides (observed in TTBK1) is 0.39.

**Figure 5:**
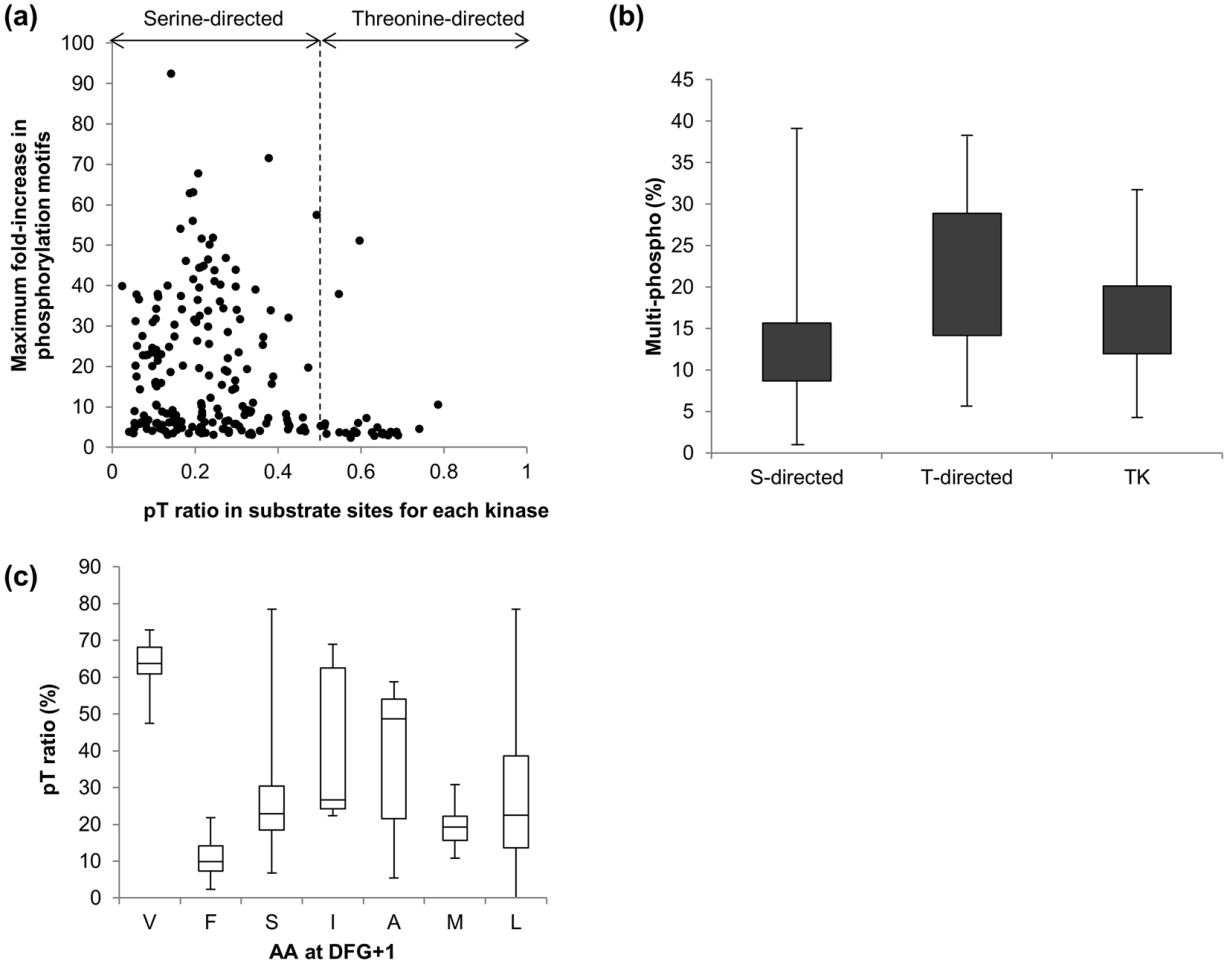
Characteristics of serine or threonine-directed kinases. (**a**) Correlation between serine/threonine preference of STKs and the maximum fold-increase in their phosphorylation motifs. (**b**) The box plot of a ratio of multiply phosphorylated peptides identified by *in vitro* kinase profiling for serine-directed, threonine-directed tyrosine kinases. (**c**) Relationship between Ser/Thr specificity and the amino acid at the +1 position of the common sequence “DFG” in the kinase domains.

Using the information about the *in vitro* substrates at the site level, we performed hierarchical cluster analysis of STKs to classify the kinases and to extract shared phosphorylation motifs (Fig. 4(a)). They were mainly separated into three clusters, of which one was STKs that had pS/T-P-containing motifs (Cluster 1). Cluster 1 consisted of most of the CMGC group, except for casein kinase, and MAP2K and MAP3K. The second cluster consisted of multiple groups, mainly containing STE, TKL, CK1 and Others (Cluster 2). Furthermore this cluster could be separated into two small clusters; *i*.*e*., STKs with threonine specificity or D/E-rich phosphorylation motifs. The STKs belonging to the third cluster, including mainly the AGC and CAMK groups, had phosphorylation motifs that were basic amino acid-rich or contained a hydrophobic amino at the +4 position (Cluster 3). Although some common structures, such as R-X-X-pS or pS/T-P, were extracted as major phosphorylation motifs for many STKs in the motif analysis, the kinases were subdivided into small groups like families or subfamilies by the cluster analysis. This result indicates that the representative phosphorylation motif for each kinase is insufficient to describe kinase profiles precisely, and substrate-based classification has more power to distinguish subtle differences between kinases, even though the substrates were acquired under *in vitro* conditions. Conventional kinase classification (the so-called kinome tree) is based on sequence and domain similarity and cellular function of kinases^2^. The substrate-based classification pattern in our study was, in general, similar to the conventional kinome tree, especially in the CMGC, AGC, CAMK and CK1 groups. However, some clear differences were observed between the classifications, such as the difference in phosphorylation preference for serine, threonine and tyrosine. STKs with tyrosine preference are marked with # in Fig. 4(a). The phosphorylation motif G-pY was extracted from LIMK1 products, and G-X-pY and S-X-pY were extracted from WEE products. These phosphorylation motifs differ from those extracted from TK products. In total, 15 STKs were selected as Tyr-directed kinases (listed in Supplementary Table S4), although some kinases such as TAK1, MOS and STLK3 show Ser/Thr/Tyr triple kinase activity. It is believed that most STKs have Ser preference rather than Thr preference, considering that the Ser/Thr ratio in the phosphoforms (5/1) is higher than that in proteins (3/2)^55^. Recently, it was reported that Haspin and Camkk2b showed an *in vitro* Thr preference with synthetic and HeLa tryptic peptides^43^. In our *in vitro* experiments, most STKs were strongly directed to Ser as expected, but MAPK, CDK and some kinases had a relatively low Ser preference. Furthermore, most of the STE group and a portion of the kinases belonging to the TKL and Others groups preferentially phosphorylated Thr (Fig. 4 and Supplementary Table S4). This is the first report to describe kinome-level profiling of the serine/threonine preference of STKs, as far as we know. Interestingly, the phosphorylation motifs of threonine-selective kinases were less significant than the pT-containing motifs of other STKs (Fig. 5(a)). These results suggested that threonine-directed kinases show lower specificity than serine-directed kinases in terms of amino acid sequence recognition. In addition, threonine-directed kinases prefer multiple phosphorylation more than serine-directed kinases and tyrosine kinases (Fig. 5(b)). Recently, a correlation between kinase Ser/Thr specificity and the amino acid residues following the common motif “DFG” in the kinase activation loop, termed “DFG + 1”, was reported by Chen *et al*.^56^. The study showed that kinases with Phe and Val at the DFG + 1 position show serine- and threonine-directed activity, respectively. The Ser/Thr preference was changed by mutation of these residues. They analyzed the Ser/Thr preference of 56 STKs *from S*. *cerevisiae* and found Ser-directed kinases have larger hydrophobic residues (Leu, Phe, and Met) at the DFG + 1 position, whereas Thr-directed kinases have branched aliphatic residues (Ile, Val). They also reported that kinases without any Ser/Thr preference have Leu or Ser at the DFG + 1. Our results based on 255 human STKs also supported their finding that all of the kinases with Phe at the DFG + 1 position showed Ser preference (Fig. 5(c)). Kinases with Met at DFG + 1 were also serine-directed, but their Ser preference was slightly weaker than that of the kinases with Phe at the DFG + 1. Kinases with Val at this position exclusively showed Thr preference. By contrast, there was no stringent rule for kinases with other amino acids at DFG + 1. The dominant group, encompassing approximately 40% of test STKs and consisting of both Ser- and Thr-directed kinases, have Leu at DFG + 1. Unlike the 56 yeast kinases, most of the human kinases with Ser at the DFG + 1 showed a weak Ser preference but not exclusively. The human kinases with Ile at the DFG + 1 showed a wide range of Ser/Thr preference, whereas Thr preference was observed in the yeast study.

The *in vitro* kinase assay data for TKs was subjected to cluster analysis in the same manner as that performed for STKs (Supplementary Fig. S4). The clustering was strongly influenced by the number of *in vitro* substrates due to the less-specific phosphorylation motifs relative to STKs. The TKs were grouped into three major clusters, and some kinase families had similar or common phosphorylation motifs. However, no additional motifs were extracted based on shared substrates.

## Discussion

Since TSAP dephosphorylation is not complete, it is important to quantify residual phosphopeptides relative to peptides phosphorylated by spiked kinases. By removing residual phosphopeptides based on the relative ratio, the peptide sequences could be used with high confidence for motif extraction. Nevertheless, the phosphorylation position within the peptide sequence should be independently considered because the fragmentation of phosphopeptides is not always sufficient for unambiguous determination of the phosphosite. In this study, we used two approaches, *i*.*e*., verification of the presence of site-determining ions and probability-based scoring using the PTM score. Although the latter method provided more stringent results, we must consider the biases caused by probability-based scoring due to the difficulty in determining phosphosite localization when MS/MS spectra contain more than one positional isomer of phosphopeptides. For this reason, we used the results obtained by the former method called site-determining ion combination (SIDIC) method^57^, in which phosphopeptides with different localization of phospho-group can be accepted when the spectrum contains different series of site-determining ions for each phosphosite.

Regarding the differences between proteins and peptides as substrate sources for *in vitro* kinase assays, it was reported that Haspin and Bmpr2 preferentially phosphorylate the N- or C-terminal positions of synthetic and HeLa tryptic peptides^43^. However, no such preference was observed in our study, in which proteins extracted from cell lysates were used as the substrate source, suggesting that peptides are not suitable as a substrate source for the extraction of phosphorylation motifs in some cases. In addition, tryptic cleavage may disrupt motif sequences, especially for basophilic motifs. Therefore, we employed proteins as the substrate source, although they were heat-treated to inactivate TSAP.

AMPK-related kinases including AMPKα1/β1/γ1, AMPKα2/β1/γ1, Nuak1, Nuak2, BRSK2, MELK, MARK1, MARK2, MARK3, MARK4, SIK and QIK were well grouped in both of the conventional kinome tree and our classification. It has been reported these kinases are regulated by phosphorylation of a T-loop Thr residue by LKB1^58^ as well as CAMKK2^59^. The parent kinase LKB1 is grouped with CAMKK1, CAMKK2 and PBK in the *in vitro* assay, whereas LKB1 is not in the same group as CAMKK1, CAMKK2 and PBK in the conventional kinome tree. This indicates that the kinase classification based on the *in vitro* assay data is more reliable to predict the physiological substrates in some cases. Note that the substrates commonly phosphorylated by the classified 4 kinases mainly contain L-X-pT or pT-X-X-G, both of which are consistent with the T-loop structure of AMPK-related kinases. Mapping of our classification to the classical kinome physiological tree indicates that kinases belonging to CMGC group, CK1 group and both of AGC and CAMK groups are exclusively proline-directed, acidophilic and basophilic, respectively (Supplementary Fig. S5). In contrast, both of TKL and Others groups consists of kinases belonging to cluster 1 and 2 in our study, and STE consists of all of clusters.

The substrate specificity of STKs was higher than that of TKs, at least in the *in vitro* assay, given that the numbers of TK substrates were generally greater than those of STK substrates and the phosphorylation motifs of TKs were less specific, although TKs are known to have limited number of substrates in physiological condition^60^. The results indicated kinase specificity observed by *in vitro* kinase profiling does not reflect their physiological specificity for the following reasons. First, cellular kinome has a wide dynamic range of expression level. Second, kinases are localized to specific subcellular compartment in general. Third, phosphatases also control phosphorylation level in physiological condition. In public database, the number of substrates widely vary according to the kinase, however it is impossible to estimate the *in vivo* specificity of kinase even if there is a difference in the number of *in vitro* substrates.

Although at least one specific substrate was observed for 348 out of 354 kinases in this study, there was few kinases which has specific consensus sequences. It means that all of the identified specific substrates are not truly specific, because of the random sampling nature of shotgun proteomics. Meanwhile, kinase families were well classified with the clustering analysis based on the *in vitro* substrates (Fig. 4). The result suggests that this dataset contain much more information rather than the simple consensus sequences. It would be possible to predict true kinase-specific substrates by building a computational model to characterize kinase substrate preferences^61^. Note that the synthetic peptides containing kinase-specific phosphosites can be used to determine the activities of protein kinases in cell lysates or tissues, based on the KAYAK strategy for instance^62^.

In this study, 175,574 *in vitro* kinase-substrate relationships including 20,669 phosphosites were identified by *in vitro* profiling of 385 human protein kinases. Among the phosphosites identified in this study, only 4,913 phosphosites are overlapped with those obtained by ultra-deep phosphoproteome study of HeLa cells reported by Shrama *et al*.^4^. It is not surprising because non-physiologically relevant substrates should be observed *in vitro* due to protein denaturation of by heating process, loss of localization and high concentration of the spiked kinase. Additional techniques and/or information such as protein-protein interaction will enable us to more accurately predict physiological kinase-substrate relationships.

Although 164 other human kinases have not yet been characterized, to our knowledge, this is currently the largest dataset relevant to human kinase-substrate relationships. We identified more than ten times larger number of human kinase-substrate relationships than those available in the most frequently used public database PhosphoSitePlus (13, 726 kinase-substrate relationships for human; last modified on Nov 30, 2018), however our dataset covered only approximately 4% of the kinase-substrate relationships published in PhosphoSitePlus, regardless of whether they were observed *in vivo* or *in vitro*. In addition, overlap of kinase-substrate relationships between our data and those obtained by using functional protein microarray^40^ is too small (Supplementary Fig. S6). One reason might be because our datasets were obtained only from the HeLa cell lysate as the substrate source. MS observability of phosphorylated peptide in this study was dependent on expression levels of substrate proteins in HeLa cells. Additional *in vitro* kinase substrate might be identified when different cell lysate is used as a substrate source since protein expression profiles vary among cell strains. Note that motifs obtained by *in vitro* kinase assay included all motifs extracted from known sites in PhosphosSitePlus when *in vitro* kinase substrates of Erk1, AKT1 and PKA were deeply identified^47^. More comprehensive profiling would be possible with additional cell lines, tissues or subcellular fractionation.

In conclusion, we profiled substrate preference of the 385 human kinases using *in vitro* assay. This very large dataset of kinase-substrate pairs is expected to be a core resource in the development of more reliable computational tools to uncover the overall phosphorylation network in cells.

## Methods

### Materials

Titanium dioxide (titania) particle (10 μm diameter) were purchased from GL Sciences (Tokyo, Japan). Thermo-sensitive alkaline phosphatase (TSAP) and modified trypsin were obtained from Promega (Madison, WI). [^2^H_2_, ^13^C]Formaldehyde was purchased from ISOTEC (Miamisburg, OH). Recombinant human protein kinases were from Carna Biosciences (Kobe, Japan), with the exception of KHS1, SGK496, NIK, CDK6/Cyclin D1, CDK9/Cyclin K, ALK1, CDK4/Cyclin D1, BARK1 and MPSK1 (Invitrogen, Carlsbad, CA) and GPRK5, FRAP, DAPK2, PKG1a, STK33, TAO1 and ULT3 (Millipore, Billerica, MA). More details on kinases are shown in Supplementary Table S5 and Supplementary Information. All other reagents were from WAKO Chemicals (Osaka, Japan).

### *In vitro* kinase assay

HeLa cells, cultured to 80% confluence in ten 15-cm diameter dishes, were suspended in 10 mL of 10 mM HEPES-NaOH (pH 7.9) containing 10 mM KCl, 1.5 mM MgCl_2_, 0.5 mM dithiothreitol, 0.5% NP-40, and protease inhibitors (Sigma). After incubation for 5 min on ice, the suspension was centrifuged at 1,500 g for 10 min, and then the buffer was exchanged with 50 mM Tris-HCl (pH 8.0) by ultrafiltration with BIOMAX-10K NMWL (Millipore). The amount of protein in the solution was measured with a BCA protein assay kit (Thermo Scientific). Thermo-sensitive alkaline phosphatase was added (10 units per mg proteins in lysate) to the concentrated cytoplasmic fraction. The reaction mixture was incubated at 37 °C for 60 min, and the phosphatase was inactivated at 75 °C for 30 min. The solution was cooled on ice for 5 min and spiked with phosphatase inhibitor cocktail (Sigma) according to the manufacturer’s manual. A small aliquot of the solution (4 μL) containing 100 μg of proteins was diluted with 16 μL of 40 mM Tris-HCl buffer (pH 7.5) containing 20 mM metal chloride, 1 mM ATP and some additives, as shown in Supplementary Table S5; phosphorylated with 0.5 μg of each protein kinase at 37 °C for 3 hours; and then heated at 95 °C for 5 min. For the control sample, 40 mM Tris-HCl buffer was spiked instead of kinase solution; the subsequent treatment was the same.

### Digestion of the kinase-treated HeLa cell cytoplasmic fraction

After *in vitro* phosphorylation by spiked kinases, Tris-HCl buffer (pH 9.0), urea and octylglycoside were added to the supernatant at final concentrations of 0.1 M, 8 M and 0.4%, respectively. Reductive alkylation and digestion were performed as previously reported^14^. In brief, the solution was reduced with 10 mM dithiothreitol for 30 min at room temperature (rt), alkylated with 50 mM iodoacetamide for 30 min at rt in the dark and digested with Lys-C (1/100 w/w) for 3 h at rt, followed by dilution with 4 volumes of 50 mM ammonium bicarbonate and digestion with trypsin (1/100 w/w) overnight at rt. These digested samples were acidified with TFA and desalted as described below.

### Desalting with an SDB-XC StageTip

Desalting using reversed phase-StageTip was performed as previously described^57,63^. In brief, a disk cut out from an SDB-XC Empore disk membrane (3 M) with a 10-gauge syringe needle was inserted into a D-1000 pipette tip (GILSON). The tip was conditioned with 100 μL of 0.1% TFA and 80% acetonitrile and then equilibrated with 100 μL of 0.1% TFA and 5% acetonitrile by centrifugation at 1000 g for 1 min. The tryptic digest corresponding to 100 μg of proteins was loaded into the tip by centrifugation at 1000 g for 5 min. The tip was washed with 100 μL of 0.1% TFA and 5% acetonitrile by centrifugation at 1000 g for 1 min. Peptides were eluted with 100 μL of 0.1% TFA and 80% acetonitrile by centrifugation at 1000 g for 1 min.

For desalting after phosphopeptide enrichment, a disk cut out from the membrane with a 16-gauge syringe needle was inserted into a pipette tip D-200 (GILSON). Desalting was performed in the same manner as described above except that the volume of solvent and loading amount of the sample were changed. In each step, 20 μL of solvent was used, and the whole sample eluted from one titania tip was loaded onto one desalting tip.

### Dimethyl labeling with formaldehyde

Dimethyl labeling of the tryptic digest with formaldehyde was performed according to the literature^64^. The desalted tryptic digest solution was concentrated in a vacuum evaporator and then dissolved in 412 μL of triethylammonium bicarbonate (pH 8.0). Sixteen microliters of 4% normal and ^2^H_2_, ^13^C-labeled formaldehyde was added to the control and kinase-treated samples, respectively. After incubation for 60 min at room temperature, the reaction mixture was cooled on ice, and then 80 μL of formic acid was added to it. The reaction was terminated by agitation for 1 min. Both solutions were mixed, concentrated in a vacuum evaporator, and subjected to phosphopeptide enrichment as described below.

### Enrichment of phosphopeptides with metal oxide chromatography

Metal oxide chromatography (MOC) using titania was performed as previously described^14^ with slight modifications. A disk cut out from a C8 Empore disk membrane (3 M) with a 20-gauge syringe needle was inserted into a 0.1–10 μL pipette tip (Eppendorf) as a frit. Then, a slurry of 0.5 mg of bulk titania beads in 10 μL of methanol was packed into the tip by centrifugation at 1000 g for 1 min. Prior to loading samples, MOC tips were equilibrated with 20 μL of 0.1% TFA and 80% acetonitrile with 300 mg/mL lactic acid as a selectivity enhancer (solution A) by centrifugation at 2000 g for 1 min. Desalted tryptic digest from a total of 100 μg of the reaction mixture was diluted with 100 μL of solution A, and a 50-μL aliquot was loaded onto the MOC tip four times by centrifugation at 1,000 g for 5 min. After successive washing with solution A and 0.1% TFA/80% acetonitrile by centrifugation at 2000 g for 1 min, the peptide was eluted with 50 μL of 0.5% piperidine by centrifugation at 1,000 g for 5 min. The eluted fraction was acidified with TFA and desalted using SDB-XC StageTips as described above. The desalted sample was concentrated in a vacuum evaporator, then resuspended in 10 μL of 0.1% TFA/80% acetonitrile for subsequent nanoLC-MS/MS analysis.

### NanoLC-MS system

NanoLC-MS/MS analyses were performed using an Orbitrap system (LTQ-Orbitrap XL, Thermo Fisher Scientific, Rockwell, IL), a Dionex Ultimate 3000 pump with FLM-3000 flow manager (Thermo Fisher Scientific) and an HTC-PAL autosampler (CTC Analytics, Zwingen, Switzerland) as previously reported^14^. In brief, ReproSil C18 materials (3 μm, Dr. Maisch, Ammerbuch, Germany) were packed into an ESI needle (150 mm length × 100 μm I.D., 6 μm opening) to prepare an analytical column^65^. The injection volume was 5 μL, and the flow rate was 500 nL/min. The mobile phases consisted of (A) 0.5% acetic acid and (B) 0.5% acetic acid and 80% acetonitrile. A three-step linear gradient of 5% to 10% B in 5 min, 10% to 40% B in 60 min, 40% to 100% B in 1 min and 100% B for 4 min was employed throughout this study. A spray voltage of 2400 V was applied. The MS scan range was *m/z* 300–1500. The top ten precursor ions were selected in the MS scan by the Orbitrap with R = 60,000 for MS/MS scans and the ion trap in automated gain control (AGC) mode, where AGC values of 5.00 × 10^5^ and 1.00 × 10^4^ were set for full MS and MS/MS, respectively. To minimize repetitive MS/MS scanning, a dynamic exclusion time was set at 20 sec with a repeat count of 1 and an exclusion list size of 500. The normalized CID was set as 35.0. A lock mass function was used to obtain constant mass accuracy during gradient analysis^66^. The MS raw data and analysis files have been deposited to the ProteomeXchange Consortium (http://proteomecentral.proteomexchange.org) via the jPOST partner repository^67^ (https://jpostdb.org) with the data set identifier PXD011366.

### Database searching

Database searching was performed as described^57^. In brief, Mass Navigator v1.2 (Mitsui Knowledge Industry, Tokyo, Japan) with the default parameters for the LTQ-Orbitrap XL was used to create peak lists based on the recorded fragmentation spectra. When the intensity of an MS/MS product ion in the peak list file was smaller than 10 or 0.1% of that of the highest peak, the ions at the noise level were removed from the peak list. The *m/z* values of the precursor isotope peaks were converted to the corresponding monoisotopic peaks when the isotope peaks were selected as the precursor ions^68^. Peptides and proteins were identified by means of automated database searching using Mascot v2.3 (Matrix Science, London) against SwissProt release 2010_11 (2-Nov-2010) with a precursor mass tolerance of 3 ppm, a fragment ion mass tolerance of 0.8 Da and strict trypsin specificity^69^ allowing for up to 2 missed cleavages. Carbamidomethylation of cysteine was set as a fixed modification, and oxidation of methionines, phosphorylation of serine, threonine and tyrosine, and [^1^H_4_, ^12^C_2_]- or [^2^H_4_, ^13^C_2_] dimethylation of amino groups at the peptide *N*-terminus and in the lysine side chain were allowed as variable modifications.

Peptides were considered identified if the Mascot score was over the 95% confidence limit based on the ‘identity’ score of each peptide and if at least three successive y- or b-ions with a further two or more y-, b- and/or precursor-origin neutral loss ions were observed, based on the error-tolerant peptide sequence tag concept^70^. The peak area of identified phosphopeptide was integrated using Mass Navigator v1.2, and then the peak area ratio for kinase-treated to control sample was calculated. Peptides for which the peak area ratio was less than or equal to 2 and incompletely modified peptides were rejected. Decoy database search using a randomized database created by a Mascot Perl script gave a 0–0.3% false-positive rate for identified peptides with these criteria. Phosphosite localization was evaluated by using an in-house Perl script to check for the presence of a site-determining ion combination^57^, and PTM scores were obtained with PhosCalc version 1.2^71^.

### Extraction of phosphorylation motifs

Using the amino acid sequences of phosphopeptides that were increased more than 2-fold with kinase reactions, all proteins containing each peptide sequence were extracted from the SwissProt database. Then, phosphoacceptor-centered 13-amino-acid sequences were extracted from the matched protein sequences, and redundant sequences were removed. Phosphorylation motifs of each kinase were extracted using Motif-x (http://motif-x.med.harvard.edu/)^51^, in which the minimum number of motif occurrences was set to 10, the significance threshold was set to 10e-6 and the IPI Human Proteome Database was used as a background.

### Classification of protein kinases

The wild-type protein kinases of which *in vitro* substrate peptides were more than 100 for STKs and 200 for TKs were classified based on *in vitro* substrate data by using Cluster 3.0^72^. The data matrix for cluster analysis was generated according to the following rules: if phosphorylation of each site was observed by each kinase reaction, the numerical value was set as 1, and if not, 0. Hierarchical cluster analysis was performed using a correlation similarity metric and centroid linkage after centering the data by subtracting mean values for columns and arrays. The clustering data were visualized as circle dendrograms were generated using iTOL^73^, ignoring branch length.

### Phosphorylation stoichiometry

The kinase reaction mixture was divided into 2 aliquots, which were labeled with normal or ^2^H_2_, ^13^C-labeled formaldehyde as described above. The stable isotope-labeled mixtures were subjected to phosphopeptide enrichment using metal oxide chromatography as described above. The two fractions were mixed and desalted with a SDB-XC stage tip and then evaporated in a vacuum concentrator. The peptides were dissolved in 50 mM Tris-HCl (pH 8.0), and 2 units of TSAP was added. After incubation at 37 °C for 60 min, the solution was desalted with a SDB-XC stage tip and evaporated in a vacuum concentrator. The residue was taken up in 10 μL of solution A for subsequent nanoLC-MSMS analysis.

## Supplementary information

Supplementary Information

Supplementary Table S1

Supplementary Table S2

Supplementary Table S3

Supplementary Table S4

Supplementary Table S5
