# Supplementary material for: Large-scale Discovery of Substrates of the Human Kinome
Source: Sci Rep. 2019 Jul 19;9:10503. doi: 10.1038/s41598-019-46385-4 (PMC6642169; doi:10.1038/s41598-019-46385-4)
Supplement: Supplementary file 1 — Supplementary Information [file 41598_2019_46385_MOESM1_ESM.pdf]

Supplementary Information

Large-scale Discovery of Substrates of the Human Kinome

Naoyuki Sugiyama[1], Haruna Imamura[1], Yasushi Ishihama[1]

[1]Graduate School of Pharmaceutical Sciences, Kyoto University, Sakyo-ku, Kyoto 606-8501, Japan

Supplemental Data

Product information of kinases in this study

- Expression systems: bacurovirus/insect cells or *E. coli*
- Sequences of kinase: full length or partial with His-tag or GST-tag
- Activity: monitored by mobility shift assay, ELISA or radioassay using substrate peptide or protein
- Purity: check by SDS-PAGE (CBB-stain)

The detailed product information of each kinase is shown in Supplementary Table S5 and available from the following URL.

Carna Biosciences

https://www.carnabio.com/english/product/protein1.html

Invitrogen

http://www.thermofisher.com/us/en/home/life-science/drug-discovery/target-and-lead-identification-and-validation/kinasebiology/kinase-proteins.html

Millipore

http://www.merckmillipore.com/JP/en/product/Kinase-Phosphatase-Products,MM_NF-C102890?CatalogCategoryID=

Supplementary Figures

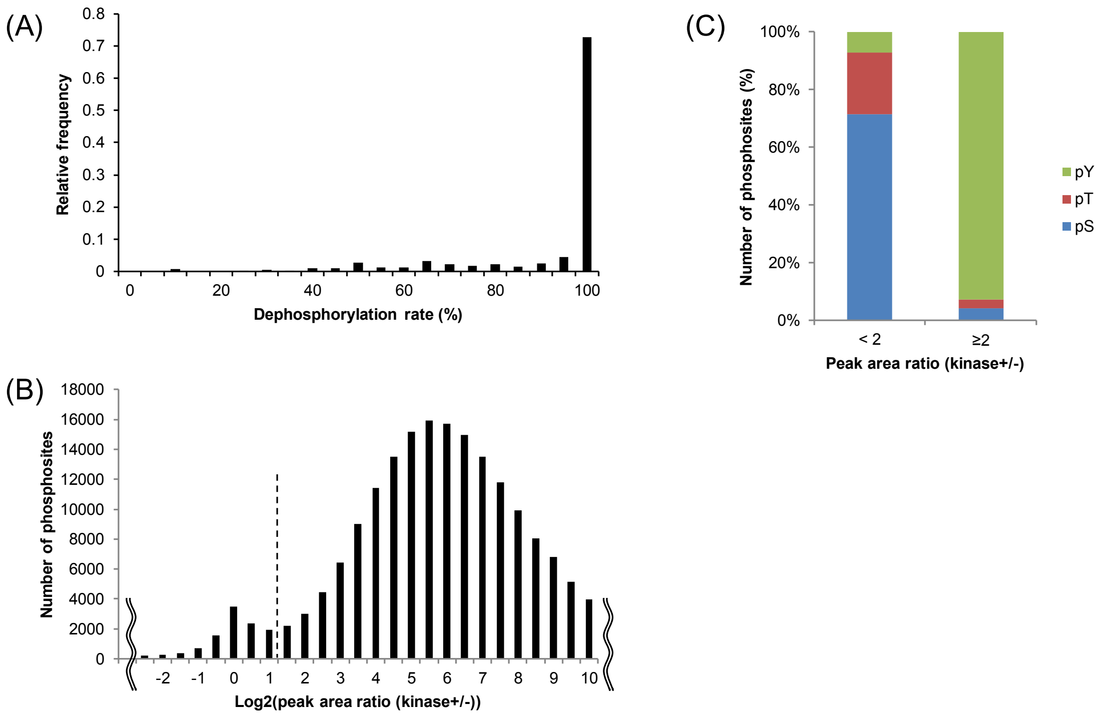

Supplementary Figure S1 Filtering of false-positive substrates by quantitative phosphoproteomics. (A) Dephosphorylation efficiency of *in vitro* phosphatase reactions. HeLa cell lysate was subjected to *in vitro* dephosphorylation reactions in the presence or absence of spiked alkaline phosphatase, followed by tryptic digestion, stable isotope-labeling with formaldehyde and enrichment of phosphopeptides with metal oxide chromatography. The dephosphorylation rate for each phosphosite was calculated based on the peak area ratio of a corresponding phosphopeptide in LC-MS analysis according to the following formula: dephosphorylation rate = $\frac{(N-D)}{N} \times 100$. N and D represent peak areas of each phosphopeptide obtained from non-treated and dephosphorylated lysate, respectively. (B) Distribution of spiked kinase-induced phosphorylation increases for each phosphosite. The peak area ratio of the kinase-spiked sample to control sample was calculated for each phosphosite. The acceptance criterion for a positive substrate, i.e., a peak area ratio of more than 2, is indicated by a dashed line.

(C) Phosphorylated serines, threonines and tyrosines obtained by *in vitro* reaction with tyrosine kinase EPHA4. Peak area ratio of each phosphosite was calculated as mentioned above.

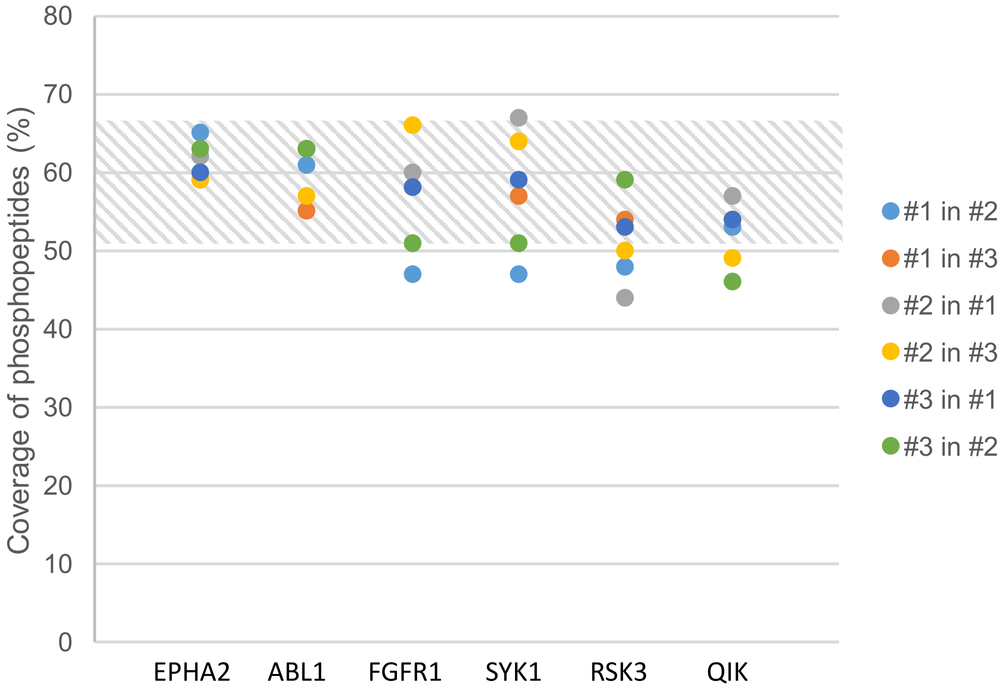

Supplementary Figure S2 Reproducibility of our *in vitro* kinase assay. Three cell cultures of HeLa were independently lysed for each kinase, and then all successive process including kinase reaction, tryptic digestion and nanoLC-MS/MS was performed. Overlaps of identified phosphopeptides between each pair of replicates are plotted for each kinase reaction. The shaded area represents a distribution of the overlaps in technical replicates of nanoLC-MS/MS analysis.

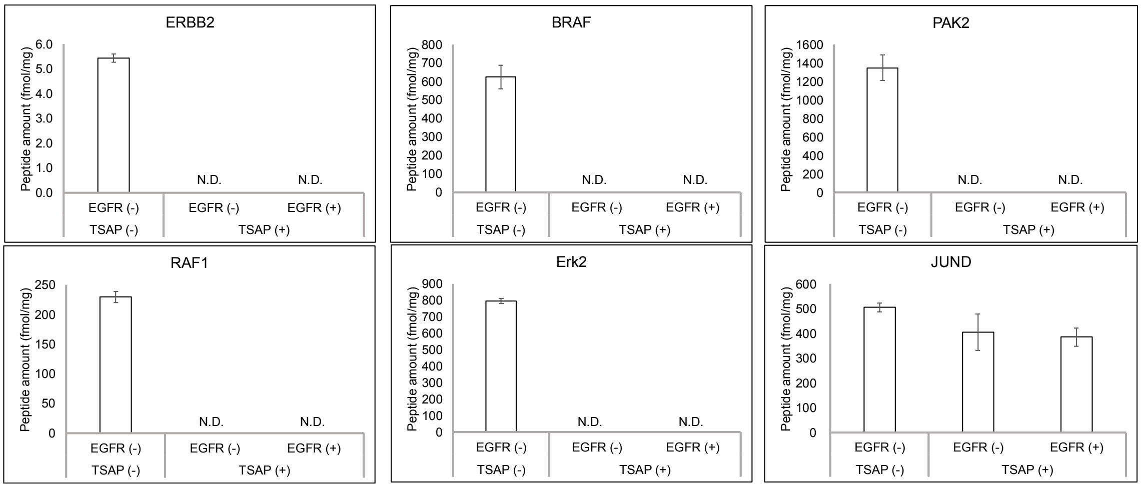

Supplementary Figure S3 Activities of endogenous kinases in the cell lysate. Phosphopeptides corresponding to regulatory sites of ERBB2, BRAF, PAK2, RAF1, and Erk2 and a known substrate of JNK1 (JUND) in the cell lysate treated with or without thermos-sensitive alkaline phosphatase (TSAP) and EGFR were quantified by selected reaction monitoring (SRM) assay for the targeted kinases activities [1]. As a positive control, a cell lysate extracted by phase transfer surfactant (PTS) [2] was treated in the same manner without addition of TSAP and EGFR.

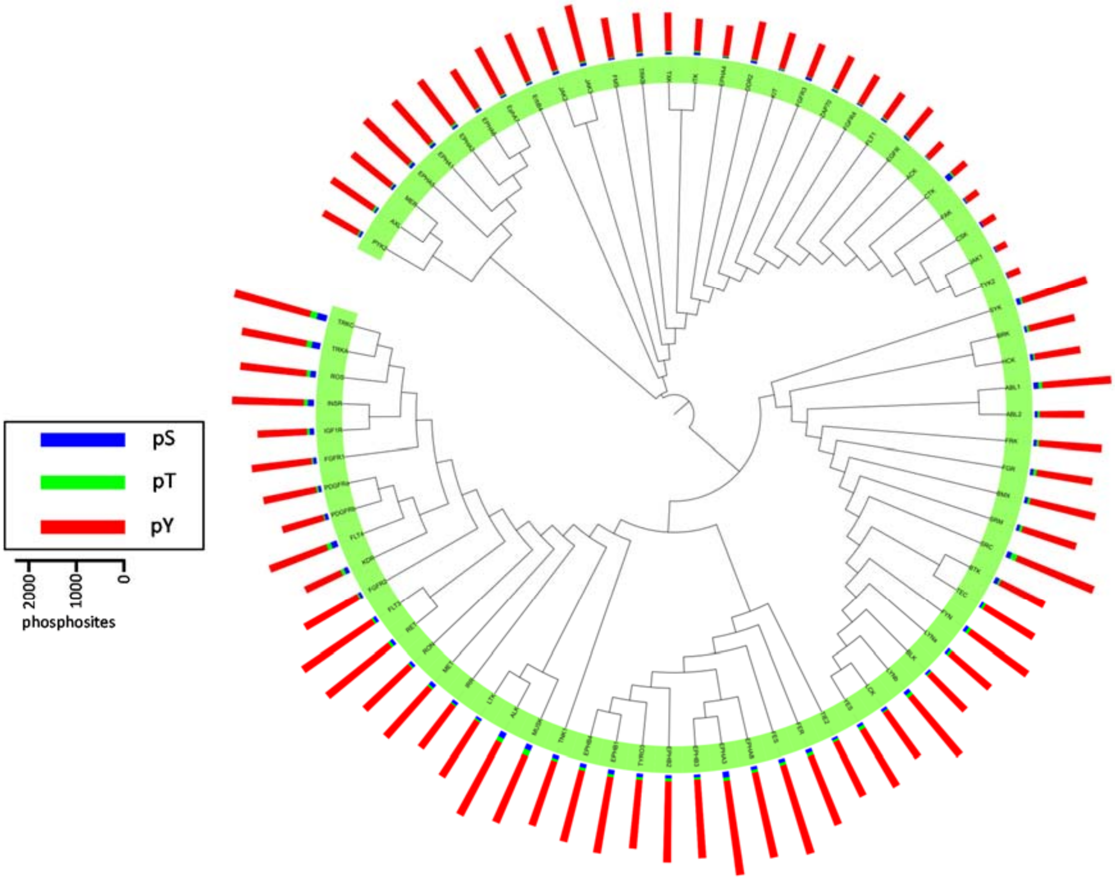

Supplementary Figure S4 Classification of tyrosine kinases based on *in vitro* substrates. A phylogenic tree was generated based on the cluster analysis.

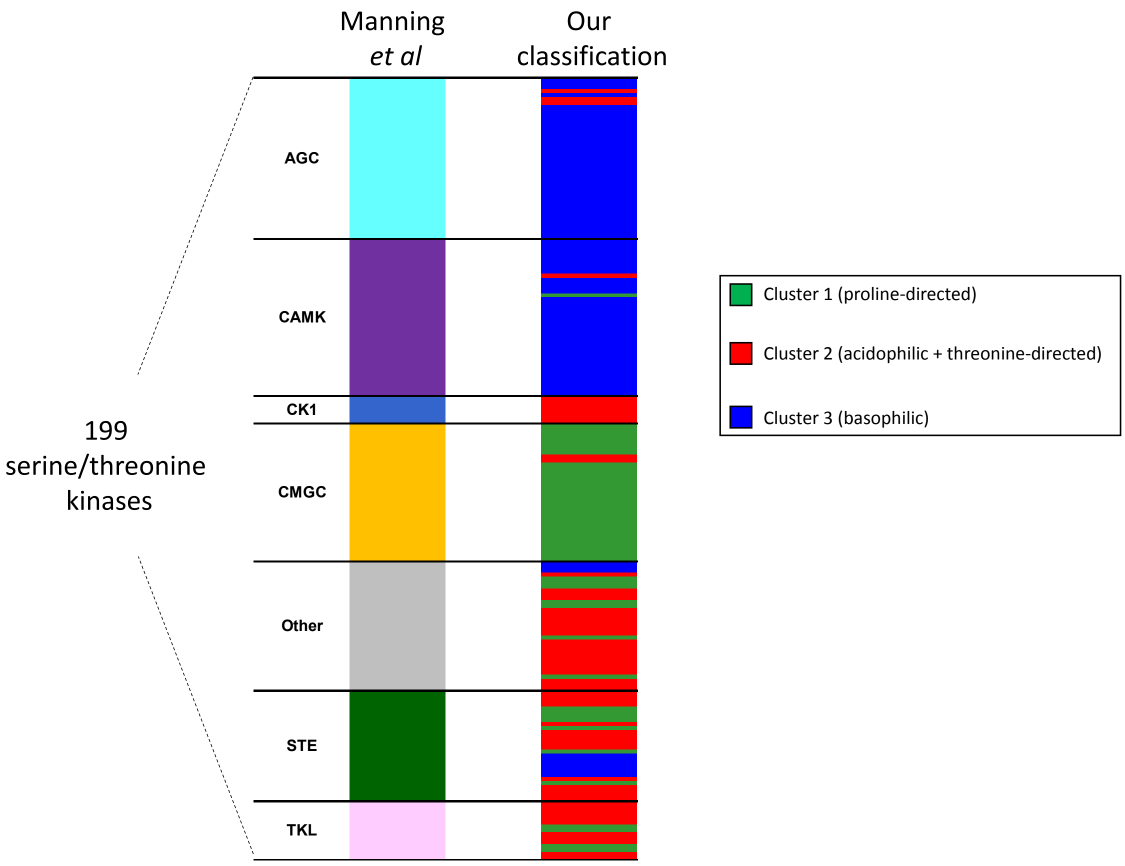

Supplementary Figure S5 Comparison between our kinase classification and the conventional kinome physiological tree [3].

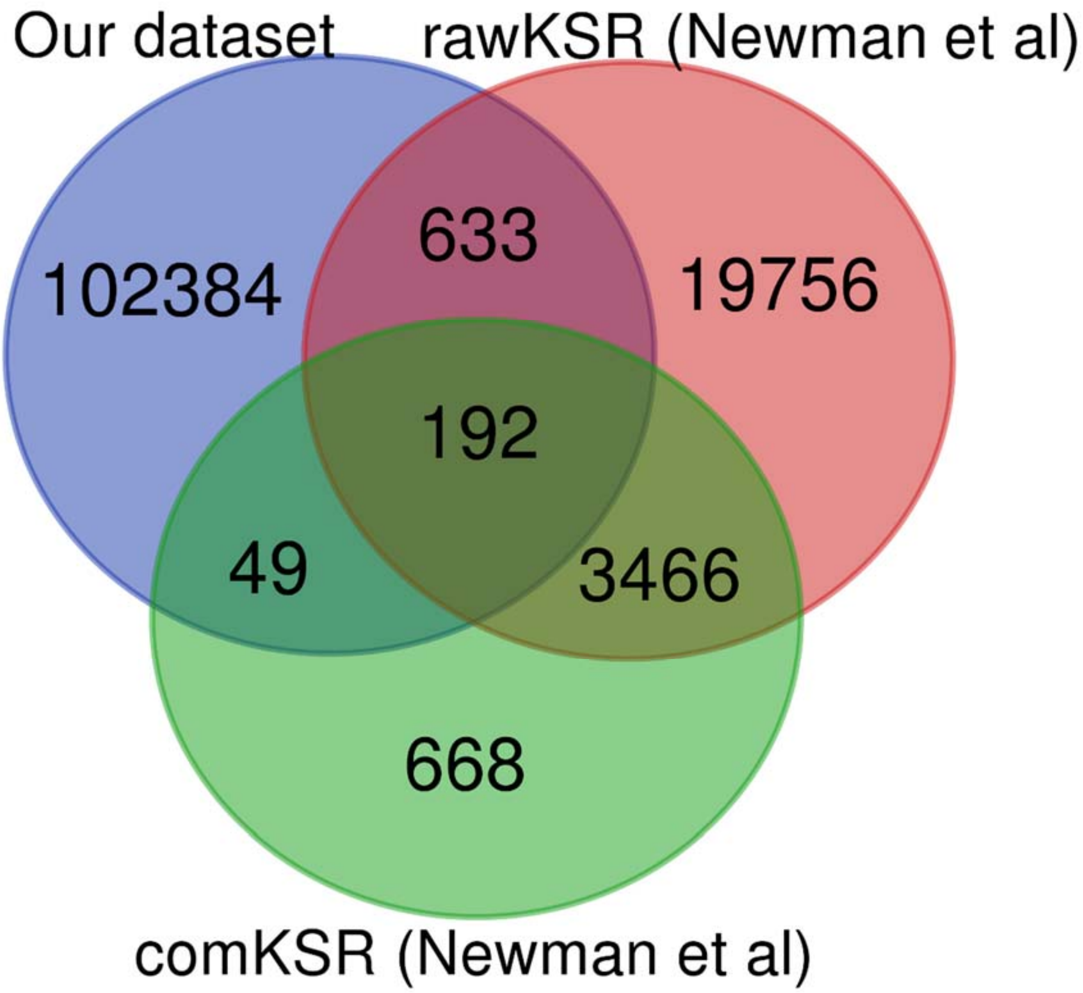

Supplementary Figure S6 .Comparison between kinase-substrate relationships in our data and those reported by Newman *et al* [4]. The rawKSR represents kinase-substrate relationships (KSR) obtained by functional protein microarray. The comKSR, which was supposed to be more physiologically relevant, consists of the refined rawKSR and known KSRs. The protein-level overlap of the KSRs was shown here.

Supplementary Tables

All of the Supplementary Tables in the following list are shown in Supplementary Dataset.

Supplementary Table S1 Phosphopeptides identified by an *in vitro* kinase assay. Phosphosites were confirmed by the presence of site-determining ions surrounding candidate sites.

Supplementary Table S2 Phosphosites identified by an *in vitro* kinase assay. Relationships between spiked recombinant protein kinases and phosphosites are shown. Phosphorylation localization was confirmed by the presence of site-determining ion combination (SIDIC)[5] or a localization probability of $P > 0.75$ based on the PTM score.

Supplementary Table S3 All phosphorylation motifs extracted from *in vitro* substrates. Phosphorylation motifs with a significant score for each kinase, classified into the same family or multi-family kinases, were extracted from the *in vitro* kinase substrates by using motif-x.

Supplementary Table S4 Threonine-directed and serine/threonine/tyrosine triple kinases. Among the STKs that phosphorylated at least 80 *in vitro* substrates, those for which more than 50% or 20% of the substrate sites were threonine or tyrosine were extracted.

Supplementary Table S5 Reaction conditions for *in vitro* profiling and product information of the recombinant kinases. Wild-type kinases which *in vitro* substrates are more than 100 for STK and 200 for TK were categorized into class "1", and other wild-type were class "2". The class 1 kinases were utilized for classification analysis.

Reference

1. Takahashi, C., Sugiyama, N. & Ishihama, Y. Selected Reaction Monitoring of Kinase Activity-Targeted Phosphopeptides. *Chromatography*, ; 10.15583/jpchrom.12019.15005 (in press).
2. Masuda, T., Tomita, M. & Ishihama, Y. Phase transfer surfactant-aided trypsin digestion for membrane proteome analysis. *J Proteome Res* **7**, 731-740 (2008).
3. Manning, G., Whyte, D.B., Martinez, R., Hunter, T. & Sudarsanam, S. The protein kinase complement of the human genome. *Science* **298**, 1912-1934 (2002).
4. Newman, R.H. et al. Construction of human activity-based phosphorylation networks. *Mol Syst Biol* **9**, 655 (2013).
5. Nakagami, H. et al. Large-scale comparative phosphoproteomics identifies conserved phosphorylation sites in plants. *Plant Physiol* **153**, 1161-1174 (2010).
